# Genome sequence of *Oceanobacillus picturae strain S1,* an halophilic bacterium first isolated in human gut

**DOI:** 10.1186/s40793-015-0081-2

**Published:** 2015-10-29

**Authors:** Jean-Christophe Lagier, Saber Khelaifia, Esam Ibraheem Azhar, Olivier Croce, Fehmida Bibi, Asif Ahmad Jiman-Fatani, Muhammad Yasir, Huda Ben Helaby, Catherine Robert, Pierre-Edouard Fournier, Didier Raoult

**Affiliations:** Unité de Recherche sur les Maladies Infectieuses et Tropicales Emergentes, UM 63, CNRS 7278, L’Institut de Recherche pour le Développement 198, Inserm 1095, Institut Hospitalo-Universitaire Méditerranée-Infection, Faculté de Médecine, Aix-Marseille Université, 27 Boulevard Jean Moulin, 13385 Marseille Cedex 5, France; Special Infectious Agents Unit, King Fahd Medical Research Center, King Abdulaziz University, Jeddah, Saudi Arabia; Department of Medical Laboratory Technology, Faculty of Applied Medical Sciences, King Abdulaziz University, Jeddah, Saudi Arabia; Department of Medical Microbiology and Parasitology, Faculty of Medicine, King Abdulaziz University, Jeddah, Saudi Arabia

**Keywords:** *Oceanobacillus picturae*, Genome, Halophilic bacteria, Human gut, Culturomics

## Abstract

*Oceanobacillus picturae* is a strain of a moderately halophilic bacterium, first isolated from a mural painting. We demonstrate, for the first time, the culture of human *Oceanobacillus picturae,* strain S1^T^, whose genome is described here, from a stool sample collected from a 25-year-old Saoudian healthy individual. We used a slightly modified standard culture medium adding 100 g/L of NaCl. We provide a short description of this strain including its MALDI-TOF spectrum, the main identification tool currently used in clinical microbiology. The 3,675,175 bp long genome exhibits a G + C content of 39.15 % and contains 3666 protein-coding and 157 RNA genes. The draft genome sequence of *Oceanobacillus picturae* has a similar size to the *Oceanobacillus kimchii* (respectively 3.67 Mb versus 3.83 Mb). The G + C content was higher compared with *Oceanobacillus kimchii* (respectively 39.15 % and 35.2 %). *Oceanobacillus picturae* shared almost identical number of genes (3823 genes versus 3879 genes), with a similar ratio of genes per Mb (1041 genes/Mb versus 1012 genes/Mb).

The genome sequencing of *Oceanobacillus picturae* strain S1 isolated for the first time in a human, will be added to the 778 genome projects from the gastrointestinal tract listed by the international consortium Human Microbiome Project.

**Electronic supplementary material:**

The online version of this article (doi:10.1186/s40793-015-0081-2) contains supplementary material, which is available to authorized users.

## Introduction

A pure culture remains essential in microbiology. Nevertheless, metagenomics studies replaced culture methods entirely with regards to the exploration of complex ecosystems. The Human Microbiome Project (HMP) is an initiative with the goal of identifying and characterizing the microorganisms which are found in association with both healthy and diseased humans. To date (25 March 2015), 778 genome projects from the gastrointestinal tract are listed by HMP [1]. Since 2012, we applied microbial culturomics (based on the multiplication of the culture condition with a rapid identification method by MALDI-TOF) in order to extend the human gut composition. Testing more than 500,000 colonies by MALDI-TOF, we isolated more than 700 different bacterial species including more than 90 new bacterial species and 180 previously known bacterial species but first isolated in humans [1]. Each new bacterial species was described by taxonogenomics, a polyphasic approach adding genome sequencing and MALDI-TOF comparison in addition to classic phenotypic characteristics [2]. In addition, in order to make the genome sequencing data available to the international scientific community, we propose the sequencing of the genomes of all bacterial species we isolated in humans, for which no genome sequencing was previously available [1]. This will facilitate the future analysis of metagenomics studies. These strains are available for the scientific community (Collection de Souches de l’Unité des Rickettsies = CSUR). Herein, we report the genome sequencing of *Oceanobacillus picturae* strain S1 isolated for the first time in humans.

The genus *Oceanobacillus* was first described by Lu et al*.* in 2001 [3] and was emended by Yumoto et al*.* in 2005 [4]. These bacteria belong to the phylum *Firmicutes*, within the family *Bacillaceae*. This genus included 17 recognized species and two subspecies. These bacteria are motile Gram-positive rods, growing obligatory aerobically or facultative anaerobically. Some of them are moderately halophilic bacteria. Bacteria from the genus *Oceanobacillus* were isolated from diverse environmental samples [5–14], including deep-sea sediment cores [3], salt fields [11], fermented shrimp paste samples [12], soy sauce production equipment [13], and traditional Korean fermented food [14]. *Oceanobacillus picturae* was originally described as *Virgibacillus picturae* in 2003 and was isolated from a mural painting from the Servilia tomb of the Roman necropolis at Carmona (Seville, Spain) [5]. Lee et al*.* reclassified this species as *Oceanobacillus picturae* in 2006 [6]. In addition to these validly published species, as a part of a large culturomics study [15], we isolated another *Oceanobacillus* species (“*Oceanobacillus massiliensis*”) from human fecal flora [16].

In this study we isolated for the *O. picturae* from humans for the first time. Strain S1 was isolated from a stool sample of a 25 year-old obese Saudi individual (BMI = 51 kg/m^2^) using a modified Columbia agar (Becton Dickinson, Pont de Claix, France) adding 100 g/L of NaCl. We described here the genome sequencing of this bacterium.

## Organism information

### Classification and features

**Fig. 1:**
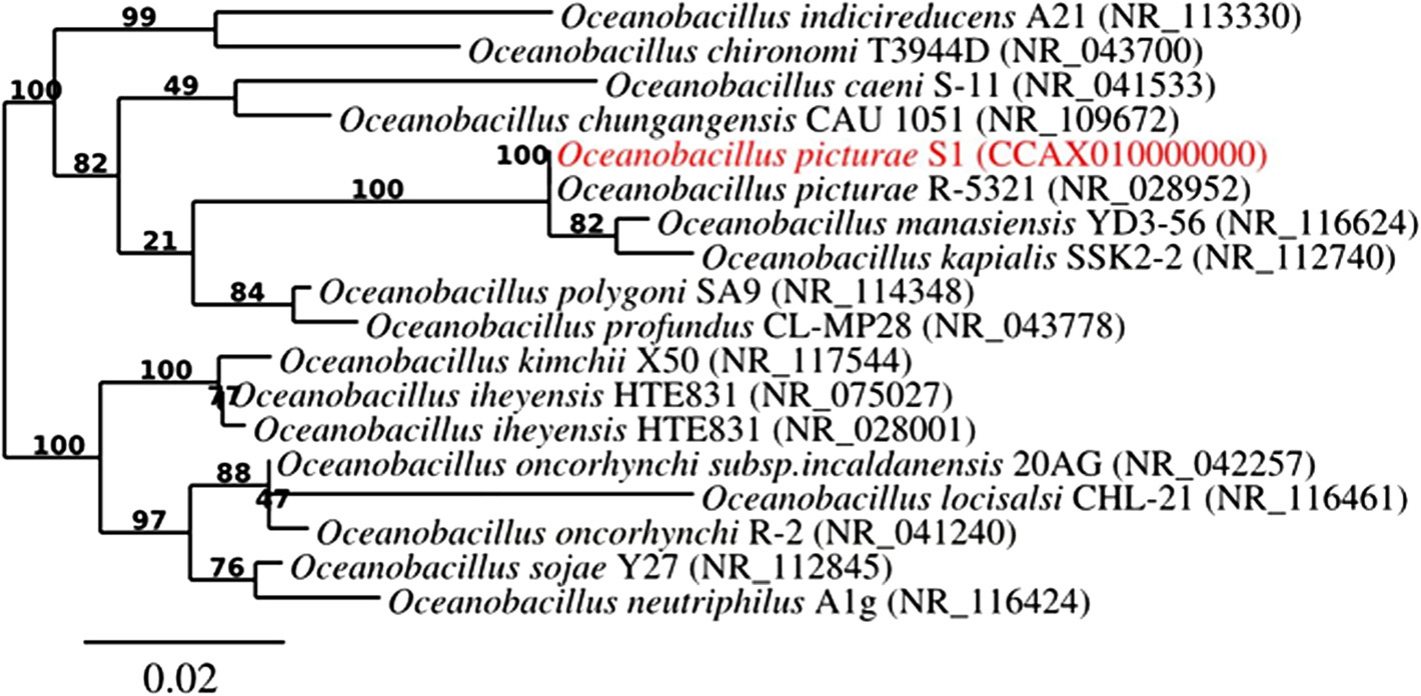
Phylogenetic tree highlighting the position of *Oceanobacillus picturae* strain S1^T^ relative to other strains of the genus *Oceanobacillus.* Strain S1^T^ (CSUR P1091 = DSM 28586) relative to other type strains within the genus *Oceanobacillus.* The strains and their corresponding GenBank accession numbers for 16S rRNA genes are (type = ^T^): *Oceanobacillus indicireducens* strain A21^T^, NR_113330, *Oceanobacillus chironomi* strain T3944D^T^, NR_043700, *Oceanobacillus caeni* strain S-11 ^T^, NR_041533, *Oceanobacillus chungangensis* strain CAU 1051^T^, NR_109672, *Oceanobacillus picturae* strain R-5321^T^, NR_028952, *Oceanobacillus manasiensis* strain YD3-56^T^, NR_116624, *Oceanobacillus kapialis* strain SSK2-2^T^, NR-112740, *Oceanobacillus polygoni* strain SA9^T^, NR_114348, *Oceanobacillus profundus* strain CL-MP28^T^, NR_043778, *Oceanobacillus kimchii* strain X50^T^, NR_117544, *Oceanobacillus iheyensis* strain HTE831^T^, NR_075027, *Oceanobacillus iheyensis* strain HTE831^T^, NR_028001, *Oceanobacillus oncorhynchi subsp.incalanensis* strain 20AG^T^, NR_042257, *Oceanobacillus locisalsi* strain CHL-21^T^, NR_116461, *Oceanobacillus sojae* strain Y27^T^, NR_112845 and *Oceanobacillus neutriphilus* strain A1g^T^, NR_116424. Phylogeny pipeline [28] was used, wherein sequences were aligned using MUSCLE [29] alignment curation by Gblocks [30] and construction of phylogenic tree performed using PhyML [31]. Numbers at the nodes are bootstrap values obtained by repeating the analysis 100 times. The scale bar represents a 2 % nucleotides sequences divergence

A stool specimen was collected from a 25-year-old Saudi obese patient. The patient gave informed and signed consent. The study and the assent procedure were approved by the Ethics Committees of the King Abdulaziz University, King Fahd medical Research Center, Saudi Arabia, under agreement number 014-CEGMR-2-ETH-P, and of the Institut Fédératif de Recherche 48, Faculty of Medicine, Marseille, France, under agreement number 09–022. The stool sample was preserved at −80 °C after collection and sent to Marseille. *O. picturae* strain S1^T^ (Table 1) was isolated in December 2013 by aerobic cultivation on a culture medium consisting of a Columbia broth culture medium (Sigma-Aldrich, Saint-Quentin Fallavier, France) modified by the addtion of 100 g/L of NaCl with a pH adjusted to 7.5. *O. picturae* strain S1 had a 16S rRNA sequence similarity of 99.8 % with the reference strain *O. picturae* strain LMG19492^T^ (Genbank accession number NR_028952) (Fig. 1). This strain was deposited in the CSUR (under number P887).

**Table 1:** Classification and general features of *Oceanobacillus picturae* strain S1^T^ according to the MIGS recommendations [17]

MIGS ID	Property	Term	Evidence code^a^
	Current classification	Domain: *Bacteria*	TAS [18]
		Phylum: *Firmicutes*	TAS [19–21]
		Class: *Bacilli*	TAS [22, 23]
		Order: *Bacillales*	TAS [24, 25]
		Family: *Bacillaceae*	TAS [26]
		Genus: *Oceanobacillus*	TAS [3]
		Species: *Oceanobacillus picturae*	IDA [5, 6]
		Type strain: S1^T^	IDA
	Gram stain	Positive	IDA
	Cell shape	Rod shaped	IDA
	Motility	Motile by polar flagellum	IDA
	Sporulation	Non sporulating	IDA
	Temperature range	Mesophile	IDA
	Optimum temperature	37 °C	IDA
	pH range; Optimum	6.5–7.5; 7	
MIGS-6.3	Salinity	0.5 to 20 %	IDA
	Optimum salinity	10 %	IDA
MIGS-22	Oxygen requirement	Aerobic	IDA
	Carbon source	Unknown	IDA
	Energy source	Unknown	IDA
MIGS-6	Habitat	Human gut	IDA
MIGS-15	Biotic relationship	Free living	IDA
	Pathogenicity	Unknown	NAS
	Biosafety level	2	IDA
MIGS-14	Isolation	Human feces	IDA
MIGS-4	Geographic location	Jeddah, Saudi Arabia	IDA
MIGS-5	Sample collection time	December 2013	IDA
MIGS-4.1	Latitude	21.422487	IDA
MIGS-4.1	Longitude	39.856184	IDA
MIGS-4.3	Depth	surface	IDA
MIGS-4.4	Altitude	0 m above sea level	IDA

^a^Evidence codes - *IDA* Inferred from Direct Assay, *TAS* Traceable Author Statement (i.e., a direct report exists in the literature), *NAS* Non-traceable Author Statement (i.e., not directly observed for the living, isolated sample, but based on a generally accepted property for the species, or anecdotal evidence). These evidence codes are from the Gene Ontology project [27]. If the evidence is IDA, then the property was directly observed for a live isolate by one of the authors or an expert mentioned in the acknowledgements

**Table 2:** Project information

MIGS ID	Property	Term
MIGS-31	Finishing quality	High quality draft
MIGS-28	Libraries used	1 mate-paired, 5-kb library
MIGS-29	Sequencing platforms	MiSeq Illumina
MIGS-31.2	Fold coverage	85×
MIGS-30	Assemblers	Spades
MIGS-32	Gene calling method	Prodigal
	Locus Tag	EMBL
	Genbank ID	CCAX00000000
	Genbank Date of Release	May, 2014
	GOLD ID	Gp0100993
	BIOPROJECT	PRJEB5522
MIGS-13	Source material identifier	CSUR P887
	Project relevance	Human gut microbiota

**Fig. 2:**
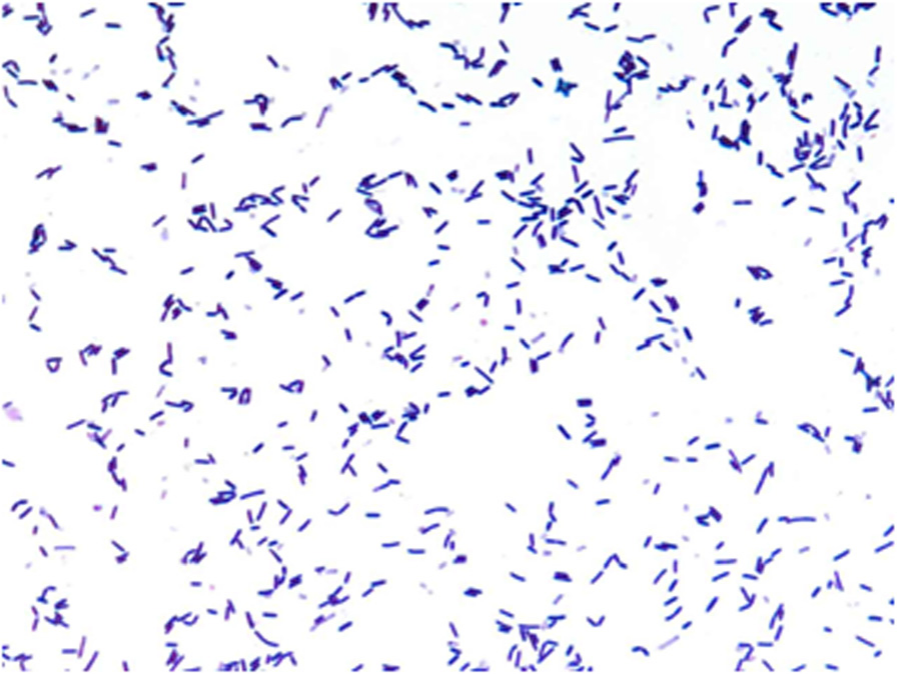
Gram staining of *Oceanobacillus picturae* strain S1^T^

**Fig. 3:**
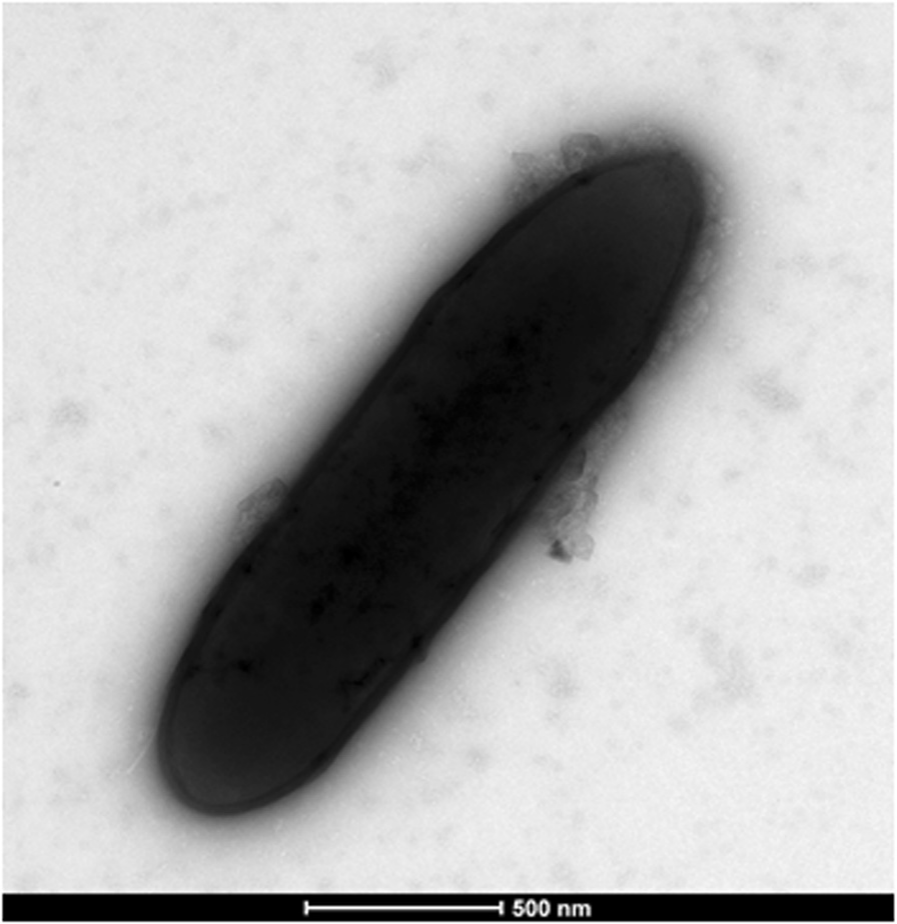
Transmission electron microscopy of *Oceanobacillus picturae* strain S1^T^, using a Morgani 268D (Philips) at an operating voltage of 60 kV. The scale bar represents 500 nm

Strain S1 colonies were observed on sheep blood agar (Biomérieux, Marcy l’Etoile, France) after 24 h of aerobic incubation at 37 °C. The colonies were greyish, 3–4 mm in diameter. Gram staining revealed Gram-positive bacilli (Fig. 2) and electron microscopy performed using a Morgani 268D (Phillips) showed rods with a mean length of 1.5 μm and a mean width of 0.5 μm (Fig. 3). Optimal growth was observed at 37 °C and strain S1 grew only under aerobe conditions.

### Extended feature descriptions

**Fig. 4:**
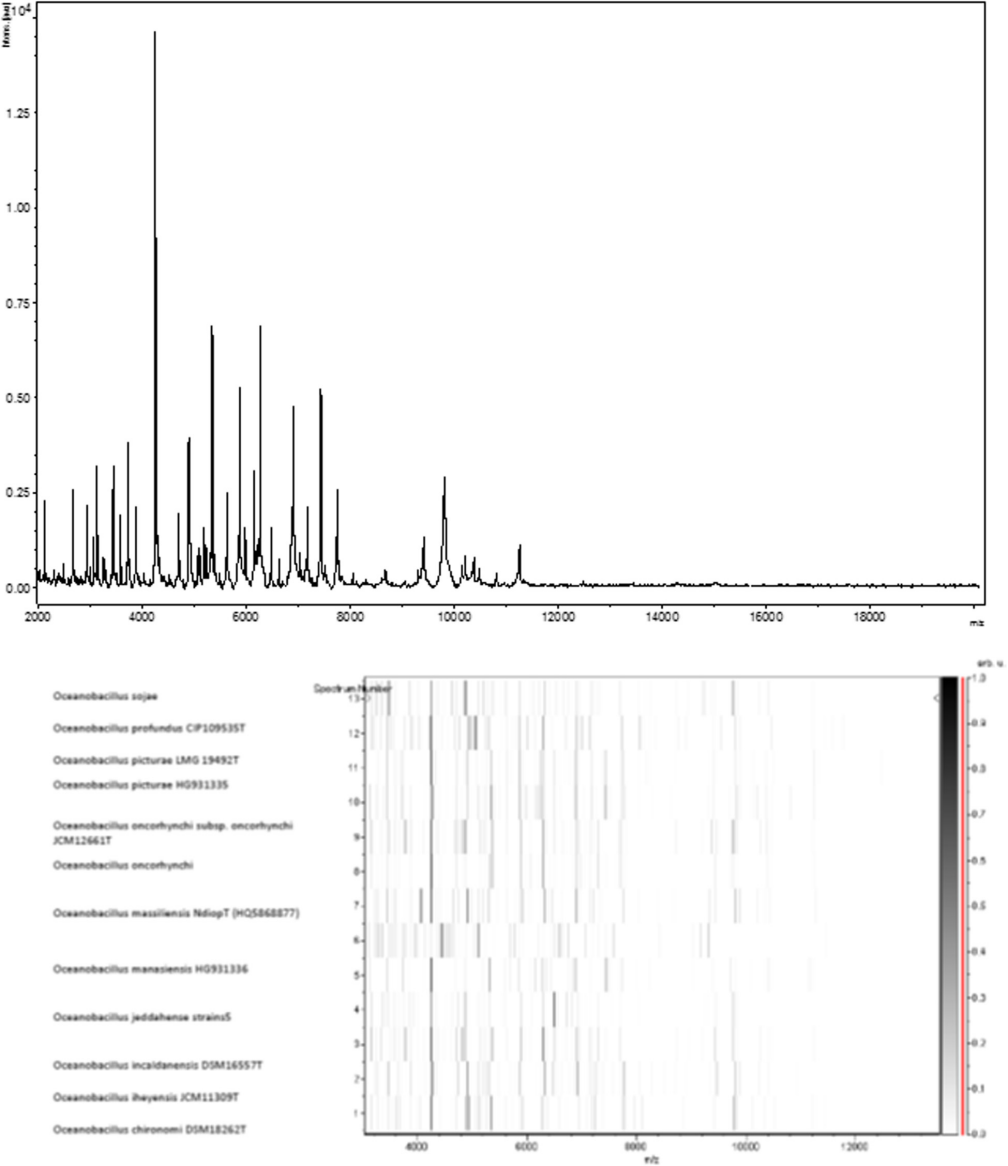
Reference mass spectrum from *Oceanobacillus picturae* strain S1^T^. Spectra from 10 individual colonies were compared and a reference spectrum was generated

We added in the description the MALDI-TOF spectra of this bacterium. Indeed, mass spectrometry has become the reference identification method in clinical microbiology [1]. Matrix-assisted laser-desorption/ionization time-of-flight (MALDI-TOF) MS protein analysis was carried out. Briefly, a pipette tip was used to pick one isolated bacterial colony from a culture agar plate and to spread it as a thin film on a MALDI-TOF target plate (Bruker Daltonics, Germany). Twelve distinct deposits were done for the strain S1 from twelve isolated colonies. After air-drying, 2 μl matrix solution (saturated solution of α-cyanohydroxycinnaminic acid in 50 % aqueous acetonitrile containing 2.5 % trifluoroacetic acid) per spot was applied. MALDI-TOF MS was conducted using the Microflex LT spectrometer (Bruker Daltonics). All spectra were recorded in the positive linear mode for the mass range of 2000 to 20,000 Da (parameter settings: ion source 1 (ISI), 20 kV; IS2, 18.5 kV; lens, 7 kV). A spectrum was obtained after 675 shots with variable laser power. The time of acquisition was between 30 s and 1 min per spot. The twelve spectra of strain S1 were imported into the MALDI BioTyper software (version 2.0, Bruker) and analyzed by standard pattern matching (with default parameter settings) against the main spectra of over 4108 bacteria including the spectra from the most closely related species including *Oceanobacillus oncorhynchi*CIP108867T, *Oceanobacillus profundus*CIP 109535T, *Oceanobacillus chironomi*CIP 109536T, *Oceanobacillus iheyensis*CIP 107618T, and *Oceanobacillus oncorhynchi* subsp. *incaldanensis*CIP 109235T, and *Oceanobacillus sojae*. In addition to these validly published species, we compared the *O. picturae* spectrum with spectra of *Oceanobacillus massiliensis* strain N’Diop and ‘*Oceanobacillus**manasiensis*’ (HG931336). The resulting score was > 2, matching with *Oceanobacillus picturae*CIP 108264 T. The identification method included the m/z from 3000 to 15,000 Da. For every spectrum, a maximum of 100 peaks were compared with spectra in the database. We added the spectrum from strain S1^T^ to our database (Fig. 4).

## Genome sequencing information

### Genome project history

The *O. picturae* genome was sequenced as part of a culturomics study aiming to isolate all bacterial species colonizing the human gut [1] (Table 2). To the best of our knowledge, *O. picturae* represent the fifth genome sequenced into the *Oceanobacillus* genus and the first genome of *O. picturae*. The genome accession number is CCAX00000000 and consists of 5 contigs without gaps. Table 3 shows the project information and its association with MIGS version 2.0 compliance.

**Table 3:** Summary of genome: 5 scaffolds

Label	Size (bp)	Topology	INSDC identifier
SCAFFOLD00001	2,198,765	Unknown	CCAX010000001
SCAFFOLD00002	704,800	Unknown	CCAX010000002
SCAFFOLD00003	480,759	Unknown	CCAX010000003
SCAFFOLD00004	282,316	Unknown	CCAX010000004
SCAFFOLD00005	8535	Unknown	CCAX010000005

### Growth conditions and genomic DNA preparation

*O. picturae* strain S1^T^ (CSUR P1091 = DSM 28586) was grown at 37 °C in an aerobic atmosphere on ten Petri dishes. The bacteria were harvested and resuspended in 4 × 100 μL of TE buffer. Then, 200 μL of this suspension was diluted in 1 mL TE buffer for lysis treatment that included a 30- min incubation with 2.5 μg/μL lysozyme at 37 °C, followed by an overnight incubation with 20 μg/μL proteinase-K at 37 °C. Extracted DNA was then purified using 3 successive phenol-chloroform extractions and ethanol precipitation at −20 °C overnight. After centrifugation, the DNA was resuspended in 160 μL TE buffer. The yield and concentration were measured by the Quant-it Picogreen kit (Invitrogen) on the Genios-Tecan fluorometer at 88.6 ng/μl.

### Genome sequencing and assembly

Genomic DNA of *Oceanobacillus picturae* was sequenced using MiSeq Technology (Illumina Inc, San Diego, CA, USA) with the mate pair strategy. The gDNA was barcoded in order to be mixed with 11 other projects with the Nextera Mate Pair sample prep kit (Illumina). The gDNA was quantified by a Qubit assay with the high sensitivity kit (Life technologies, Carlsbad, CA, USA) to 40.5 ng/μl. The mate pair library was prepared with 1 μg of genomic DNA using the Nextera mate pair Illumina guide. The genomic DNA sample was simultaneously fragmented and tagged with a mate pair junction adapter. The profile of the fragmentation was validated on an Agilent 2100 BioAnalyzer (Agilent Technologies Inc, Santa Clara, CA, USA) with a DNA 7500 labchip. The DNA fragments ranged in size from 1 kb up to 10 kb. No size selection was performed and only 14 ng of tagmented fragments were circularized. The circularized DNA was mechanically sheared to small fragments with an optimum at 696 bp on the Covaris device S2 in microtubes (Covaris, Woburn, MA, USA). The library profile was visualized on a High Sensitivity Bioanalyzer LabChip (Agilent Technologies Inc, Santa Clara, CA, USA). The libraries were normalized at 2 nM and pooled. After a denaturation step and dilution at 10pM, the pool of libraries was loaded onto the reagent cartridge and then onto the instrument along with the flow cell. Automated cluster generation and sequencing runs were performed in a single 42-h run in a 2x251-bp. Total information of 4.7 Gb was obtained from a 488 K/mm2 cluster density with a cluster passing quality control filters of 97.2 % (9,590,000 clusters). Within this run, the index representation for *O. picturae* was determined to be 11.16 %. Illumina reads were trimmed using Trimmomatic [32], then assembled through Spades software [33, 34]. Contigs obtained were combined together by SSpace [35] and Opera software v1.2 [36] helped by GapFiller V1.10 [37] to reduce the set. Some manual refinements using CLC Genomics v7 software cCLC bio, Aarhus, Denmark) and homemade tools improved the genome. Finally, the draft genome of *Oceanobacillus picturae* consists of 5 contigs without gaps.

### Genome annotation

Non-coding genes and miscellaneous features were predicted using RNAmmer [38], ARAGORN [39], Rfam [40], PFAM [41], Infernal [42]. Coding DNA sequences (CDSs) were predicted using Prodigal [43] and functional annotation was achieved using BLAST+ [44] and HMMER3 [45] against the UniProtKB database [46].

## Genome properties

**Fig. 5:**
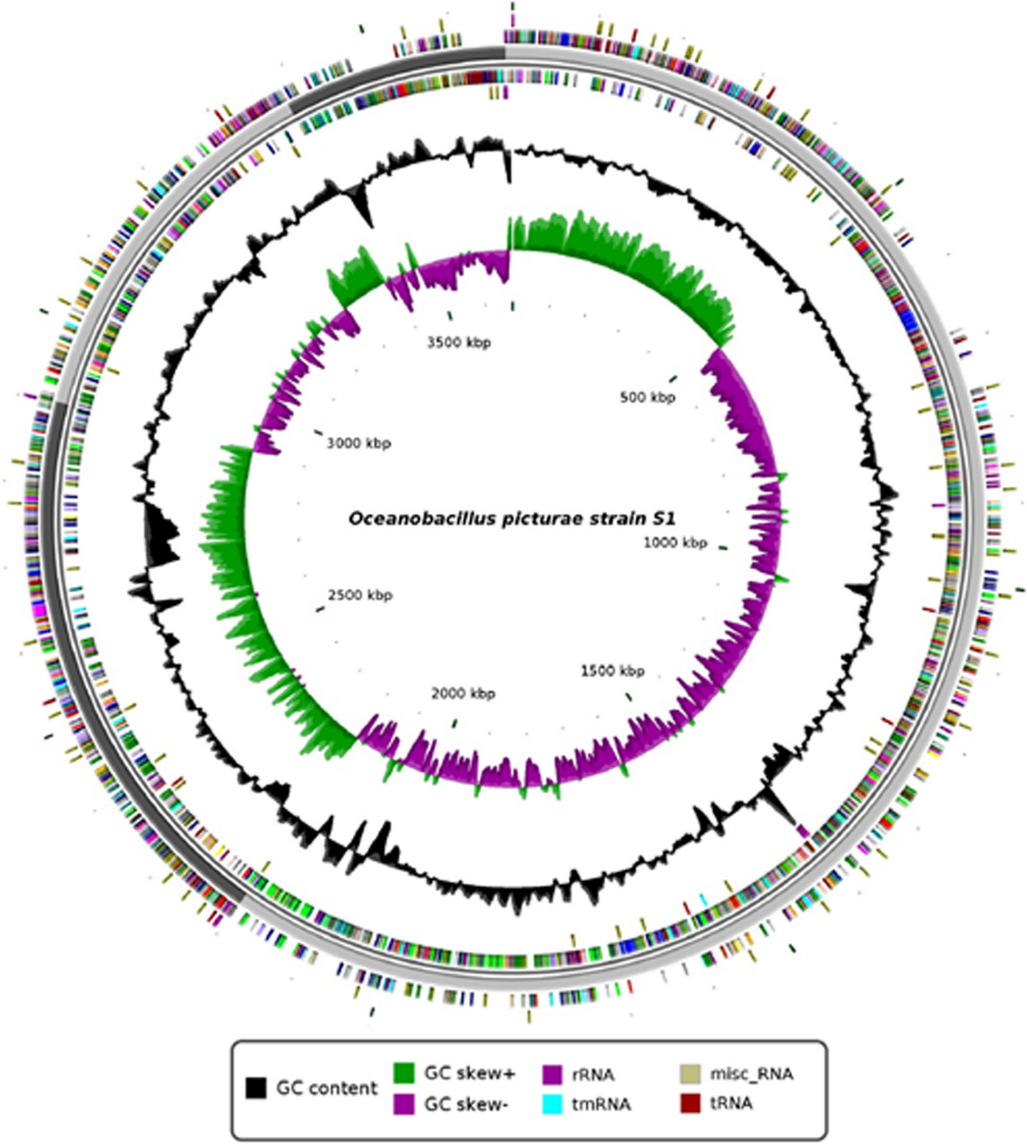
Circular representation of the *Oceanobacillus picturae* genome. Circles from the center to the outside: GC screw (green/purple), GC content (black), tRNA (dark red), rRNA (purple), tmRNA (blue), miscellaneous RNA (beige) on forward strand, genes on forward strand colored by COGs categories, scaffolds in alternative grays, genes on reverse strand colored by COGs, tRNA (dark red), rRNA (purple), tmRNA (blue), miscellaneous RNA (beige) on reverse strand

The genome of *Oceanobacillus picturae* contained 3,675,175 bp with a G + C content of 39.15 % (Fig. 5, Tables 3 and 4). The genome was shown to encode at least 157 predicted RNA including 14 rRNA, 31 tRNA, 1 tmRNA and 111 miscellaneous RNA. In addition, 3666 genes were identified, representing a coding capacity of 3,125,691 bp (coding percentage: 85.05 %). Among these genes, 269 (7.34 %) were found as putative proteins and 891 (24.3 %) were assigned as hypothetical proteins. Moreover, 3595 genes matched at least one sequence in Clusters of Orthologous Groups database [47] with BLASTP default parameters. The properties and the statistics of the genome are summarized in Tables 4 and 5. The distribution of genes into COGs functional categories is presented in Table 6 [48].

**Table 4:** Nucleotide content and gene count levels of the genome

Attribute	Value	% of total
Genome size (bp)	3,675,175	100
DNA coding (bp)	3,125,691	85.05
DNA G + C (bp)	1,438,948	39.15
DNA scaffolds	3,675,175	100
Total genes	3823	100
Protein coding genes	3666	85.05
RNA genes	157	4.11
Pseudo genes	269	7
Genes in internal clusters	1751	45.8
Genes with function prediction	2775	72.59
Genes assigned to COGs	3595	98.06
Genes with Pfam domains	183	4.79
Genes with signal peptides	229	5.99
Genes with transmembrane helices	1074	28.09
CRISPR repeats	0	0

**Table 5:** Number of genes associated with the 25 general COG functional categories

Code	Value^a^	% of total	Description
J	197	5.37	Translation, ribosomal structure and biogenesis
A	4	0.12	RNA processing and modification
K	263	7.18	Transcription
L	173	4.73	Replication, recombination and repair
B	4	0.12	Chromatin structure and dynamics
D	56	1.54	Cell cycle control, cell division, chromosome partitioning
Y	1	0.02	Nuclear structure
V	71	1.93	Defense mechanisms
T	166	4.53	Signal transduction mechanisms
M	193	5.27	Cell wall/membrane biogenesis
N	81	2.2	Cell motility
Z	4	0.12	Cytoskeleton
W	0	0.0	Extracellular structures
U	75	2.05	Intracellular trafficking and secretion, and vesicular transport
O	114	3.12	Posttranslational modification, protein turnover, chaperones
C	181	4.95	Energy production and conversion
G	240	6.56	Carbohydrate transport and metabolism
E	298	8.12	Amino acid transport and metabolism
F	91	2.48	Nucleotide transport and metabolism
H	113	3.07	Coenzyme transport and metabolism
I	103	2.8	Lipid transport and metabolism
P	214	5.84	Inorganic ion transport and metabolism
Q	62	1.68	Secondary metabolites biosynthesis, transport and catabolism
R	475	12.97	General function prediction only
S	484	13.2	Function unknown

^a^The total is based on the total number of protein coding genes in the annotated genome

### Genome comparison with *O. picturae* with *O. kimchii*

We performed a brief comparison of *Oceanobacillus picturae* strain S1 genome sequence against *Oceanobacillus kimchii* X50 (NZ_CM001792), which is currently the closest available sequenced genome based on rRNA 16S identity. The draft genome sequence of *Oceanobacillus picturae* has a similar size to the *Oceanobacillus kimchii* (respectively 3.67 Mb versus 3.83 Mb). The G + C content was higher as compared to *Oceanobacillus kimchii* (respectively 39.15 and 35.2 %). *Oceanobacillus picturae* shared an almost identical number of genes (3823 genes versus 3879 genes), with a similar ratio of genes per Mb (1041 genes/Mb versus 1012 genes/Mb). Additional file 1: Table S1 presents the difference in gene number (percentage) related to each COG categories between *O. pictuare* and *O. kimchii**.* The proportion of COGs is very similar between the two species. The maximum difference is related to the COG “Carbohydrate transport and metabolism” which does not exceed 1.15 %. Additional file 2: Table S2 presents the associated MIGS records.

## Conclusion

*Oceanobacillus picturae* strain S1 was the first strain of this bacterial species isolated from the human gut. The G + C % content of the genome was 39.15 %. The 16S rRNA and genome sequences were deposited in EMBL/EBI database under accession numbers HG931335 and CCAX00000000 respectively.

## Additional files

Additional file 1: Table S1. Percentage of genes associated with the 25 general COG functional categories for *O. picturae* and *O. kimchii X50.* (DOC 45 kb) Additional file 2: Table S2. Associated MIGS record. (DOC 70 kb)

## Abbreviations

CSUR: Collection de souches de l’Unité des Rickettsies
DSM: Deutsche Sammlung von Mikroorganismen
