# Supplementary material for: Genome sequence of Oceanobacillus picturae strain S1, an halophilic bacterium first isolated in human gut
Source: Stand Genomic Sci. 2015 Oct 29;10:91. doi: 10.1186/s40793-015-0081-2 (PMC4627390; doi:10.1186/s40793-015-0081-2)
Supplement: Additional file 1: Table S1. — Percentage of genes associated with the 25 general COG functional categories for O. picturae and O. kimchii X50. (DOC 45 kb) [file 40793_2015_81_MOESM1_ESM.doc]

**Table** [**S1**](http://www.ebi.ac.uk/chebi/chebiOntology.do?treeView=true&chebiId=CHEBI:15138)**:** Percentage of genes associated with the 25 general COG functional categories for *O. picturae* and *O. kimchii X50* .

| **Code** | **Description** | [***O. picturae***](http://dx.doi.org/10.1601/nm.9805)  % of total | [***O. kimchii***](http://dx.doi.org/10.1601/nm.21354)  % of total | **Difference (in %)** |
| --- | --- | --- | --- | --- |
| J | Translation, ribosomal structure and biogenesis | 5.37 | 4.79 | 0.58 |
| A | RNA processing and modification | 0.12 | 0.14 | -0.02 |
| K | Transcription | 7.18 | 7.55 | -0.37 |
| L | Replication, recombination and repair | 4.73 | 4.06 | 0.67 |
| B | Chromatin structure and dynamics | 0.12 | 0.17 | -0.05 |
| D | Cell cycle control, cell division, chromosome partitioning | 1.54 | 1.35 | 0.19 |
| Y | Nuclear structure | 0.02 | 0.02 | 0 |
| V | Defense mechanisms | 1.93 | 1.78 | 0.15 |
| T | Signal transduction mechanisms | 4.53 | 3.99 | 0.54 |
| M | Cell wall/membrane biogenesis | 5.27 | 4.77 | 0.5 |
| N | Cell motility | 2.2 | 1.83 | 0.37 |
| Z | Cytoskeleton | 0.12 | 0.09 | 0.03 |
| W | Extracellular structures | 0.0 | 0 | 0 |
| U | Intracellular trafficking and secretion, and vesicular transport | 2.05 | 1.95 | 0.1 |
| O | Posttranslational modification, [protein](http://www.ebi.ac.uk/chebi/chebiOntology.do?treeView=true&chebiId=CHEBI:36080) turnover, chaperones | 3.12 | 2.9 | 0.22 |
| C | Energy production and conversion | 4.95 | 5.24 | -0.29 |
| G | Carbohydrate transport and metabolism | 6.56 | 7.71 | -1.15 |
| E | Amino acid transport and metabolism | 8.12 | 8.14 | -0.02 |
| F | Nucleotide transport and metabolism | 2.48 | 2.56 | -0.08 |
| H | Coenzyme transport and metabolism | 3.07 | 3.46 | -0.39 |
| I | Lipid transport and metabolism | 2.8 | 2.97 | -0.17 |
| P | Inorganic ion transport and metabolism | 5.84 | 5.86 | -0.02 |
| Q | Secondary metabolites biosynthesis, transport and catabolism | 1.68 | 1.99 | -0.31 |
| R | General function prediction only | 12.97 | 13.05 | -0.08 |
| S | Function unknown | 13.2 | 13.62 | -0.42 |
