# Supplementary material for: Genome sequence of Oceanobacillus picturae strain S1, an halophilic bacterium first isolated in human gut
Source: Stand Genomic Sci. 2015 Oct 29;10:91. doi: 10.1186/s40793-015-0081-2 (PMC4627390; doi:10.1186/s40793-015-0081-2)
Supplement: Additional file 2: Table S2. — Associated MIGS record. (DOC 70 kb) [file 40793_2015_81_MOESM2_ESM.doc]

**Table** [**S2**](http://www.ebi.ac.uk/chebi/chebiOntology.do?treeView=true&chebiId=CHEBI:29387)**.** Associated MIGS record

| **MIGS-ID** | field name | Description |
| --- | --- | --- |
| **MIGS-1** | Submit to INSDC/Trace archives | Not reported |
| **1.1** | PID | Not reported |
| **1.2** | Trace Archive | Not reported |
| **MIGS-2** | MIGS CHECK LIST TYPE | Not reported |
| **MIGS-3** | Project Name | *Oceanobacillus picturae strain* [*S1*](http://www.ebi.ac.uk/chebi/chebiOntology.do?treeView=true&chebiId=CHEBI:15138) |
| **MIGS-4** | Geographic Location | Jeddah, Saudi Arabia |
| **4.1** | Latitude | 21.422487 |
| **4.2** | Longitude | 39.856184 |
| **4.3** | Depth | Surface |
| **4.4** | Altitude | 0 m above sea level |
| **MIGS-5** | Time of Sample collection | December 2013 |
| **MIGS-6** | Habitat (EnvO) | Human gut |
| **6.1** | Temperature | 37°C |
| **6.2** | pH | Not reported |
| **6.3** | Salinity | Moderately halophilic |
| **6.4** | Chlorophyll | Not reported |
| **6.5** | Conductivity | Not reported |
| **6.6** | light intensity | Not reported |
| **6.7** | dissolved organic carbon (DOC) | Not reported |
| **6.8** | Current | Not reported |
| **6.9** | atmospheric data | Not reported |
| **6.10** | Density | Not reported |
| **6.11** | Alkalinity | Not reported |
| **6.12** | dissolved oxygen | Not reported |
| **6.13** | particulate organic carbon (POC) | Not reported |
| **6.14** | [Phosphate](http://www.ebi.ac.uk/chebi/chebiOntology.do?treeView=true&chebiId=CHEBI:26020) | Not reported |
| **6.15** | [Nitrate](http://www.ebi.ac.uk/chebi/chebiOntology.do?treeView=true&chebiId=CHEBI:17632) | Not reported |
| **6.16** | Sulfates | Not reported |
| **6.17** | Sulfides | Not reported |
| **6.18** | primary production | Not reported |
| **MIGS-7** | Subspecific genetic lineage | Not reported |
| **MIGS-9** | Number of replicons | Not reported |
| **MIGS-10** | Extrachromosomal elements | Not reported |
| **MIGS-11** | Estimated Size | 3,675,175 |
| **MIGS-12** | Reference for biomaterial or Genome report | Not reported |
| **MIGS-13** | Source material identifiers | Not reported |
| **MIGS-14** | Known Pathogenicity | No |
| **MIGS-15** | Biotic Relationship | Not reported |
| **MIGS-16** | Specific Host | Not reported |
| **MIGS-17** | Host specificity or range (taxid) | Not reported |
| **MIGS-18** | Health status of Host | Obese individual |
| **MIGS-19** | Trophic Level | Not reported |
| **MIGS-22** | Relationship to Oxygen | aerobic |
| **MIGS-23** | Isolation and Growth conditions | Columbia broth with addition of 100 g/L [NaCl](http://www.ebi.ac.uk/chebi/chebiOntology.do?treeView=true&chebiId=CHEBI:26710) |
| **MIGS-27** | [Nucleic acid](http://www.ebi.ac.uk/chebi/chebiOntology.do?treeView=true&chebiId=CHEBI:33696) preparation | phenol-chloroform extraction |
| **MIGS-28** | Library construction | Illumina MiSeq mate-paired library |
| **28.1** | Library size | ~5 kb |
| **28.2** | Number of reads | Not reported |
| **28.3** | Vector | Not reported |
| **MIGS-29** | Sequencing method | MiSeq Illumina |
| **MIGS-30** | Assembly | Spades |
| **30.1** | Assembly method | Not reported |
| **30.2** | estimated error rate | Not reported |
| **30.3** | method of calculation | Multi k-mers assembly |
| **MIGS-31** | Finishing strategy | High quality draft |
| **31.1** | Status | Draft genome, unfinished |
| **31.2** | Coverage | 85 |
| **31.3** | Contigs | 5 |
| **MIGS-32** | Relevant SOPs | Not reported |
| **MIGS-33** | Relevant e-resources | Not reported |
